# Cardiac Tamponade: An Initial Manifestation of Diffuse Large B Cell Lymphoma

**DOI:** 10.7759/cureus.60418

**Published:** 2024-05-16

**Authors:** FNU Arty, Suyesh Chinchanikar, Mahrukh A Khan, Nuri Tamanna, David Anwar, Shazia M Shah

**Affiliations:** 1 Internal Medicine, Monmouth Medical Center, Long Branch, USA; 2 Medicine, Monmouth Medical Center, Long Branch, USA; 3 Internal Medicine, St Joseph's Health Center and Rome Health, New York City, USA; 4 Cardiology, Monmouth Medical Center, Long Branch, USA; 5 Internal Medicine, Rutgers Health/Monmouth Medical Center, Long Branch, USA

**Keywords:** extranodal disease, pericardiocentesis, diffuse large b cell lymphoma, non hodgkin lymphoma, cardiac tamponade

## Abstract

Pericardial effusion, a clinical condition characterized by an abnormal accumulation of fluid in the pericardial cavity, has multiple etiological factors. One of the prominent causes is malignant effusion.

The patient is a 69-year-old female with a past medical history of Crohn’s disease, melanoma status post-resection, and osteoarthritis. She presented with complaints of abdominal discomfort, shortness of breath on exertion, and lower extremity swelling for the past 2-3 days. She was recently discharged four days before this visit after being treated for a viral infection. A physical examination was significant for tachycardia, muffled heart sounds, abdominal distention, and bilateral lower extremity swelling. Labs were in the normal range except for elevated liver enzymes and sodium of 130 mmol/L. A chest X-ray revealed a small bilateral effusion; a bedside echocardiogram showed an ejection fraction greater than 70% and a large pericardial effusion >2 cm, consistent with cardiac tamponade. Emergent pericardiocentesis was performed with the drainage of 250 milliliters of hemorrhagic fluid, which was sent for diagnostic studies. Post-procedure echo on the next day showed an EF of 35-40% and no recurrent pericardial effusion. The workup for connective tissue disease was negative except for elevated antinuclear antibodies (ANA). CT of the abdomen and pelvis revealed gastric wall thickening with no solid organ mass. Her pericardial fluid studies were consistent with exudative etiology and positive for atypical lymphoid cells, leading to the diagnosis of diffuse large B-cell lymphoma.

Diffuse large B-cell lymphoma is the most common type of non-Hodgkin lymphoma. Malignant pericardial effusion is common due to solid organ malignancy; however, it is rare with diffuse large B cell lymphoma (DLBCL). We present an intriguing case where pericardial effusion was the precursor to the eventual diagnosis of DLBCL, highlighting the complexity and diverse manifestations of this lymphoma subtype.

## Introduction

Pericardial effusion, a clinical condition characterized by an abnormal accumulation of fluid in the pericardial cavity, has multiple etiological factors. One of the prominent factors is solid tumor metastasis. Among the various malignancies, diffuse large B-cell lymphoma (DLBCL) stands out as the most prevalent type of non-Hodgkin lymphoma (NHL), impacting approximately seven individuals per 100,000 annually [1]. Globally, DLBCL accounts for 25-30% of all NHL cases, exhibiting a male predominance and typically presenting at a median age of 64 years. DLBCL is notorious for its aggressive nature, commonly manifesting as an enlarged lymph node or a rapidly enlarging mass situated in the neck, abdomen, or mediastinum. This may be accompanied by B symptoms such as fever, night sweats, and weight loss. Notably, extranodal occurrences are observed in about 50% of DLBCL patients, with the most frequent sites being the stomach, gastrointestinal tract, and skin. Although mediastinal NHL can lead to pleural effusions in approximately 20-30% of cases, the progression to cardiac tamponade remains an exceptionally rare clinical event [2].

We present an intriguing case where recurrent pericardial effusion was the precursor to the eventual diagnosis of DLBCL, highlighting the complexity and diverse manifestations of this lymphoma subtype.

## Case presentation

The patient is a 69-year-old female with a past medical history of Crohn’s disease, melanoma status post-resection, and osteoarthritis. She presented with complaints of abdominal discomfort, shortness of breath with exertion, and lower extremity swelling for 2-3 days before admission. She was admitted four days before this with a presumed viral infection and was treated for hyponatremia and dehydration. After discharge, she developed abdominal discomfort, shortness of breath, and lower extremity swelling for the past 2-3 days. The patient conferred with her primary care physician and was advised to present to the hospital. A review of the system was positive for shortness of breath, abdominal distention, lower extremity swelling, arthralgia, and fatigue. In the emergency department, she was hemodynamically stable, saturating well on room air. Physical examination was significant for tachycardia, muffled heart sounds, abdominal distention, and lower extremity edema, without jugular vein distention (JVD).

Labs revealed sodium at 130 millimoles per liter (mmol/L), mildly elevated liver enzymes, brain natriuretic peptide (BNP) at 109 picograms per milliliter (pg/mL), and troponin negative, as shown in Table 1. 

**Table 1:** Showing abnormal lab results on admission

Lab value	Pt’s value	Normal range
Sodium	130 mmol/L	135-145 mmol/L
Alanine aminotransferase (ALT)	168 U/L	10-43 U/L
Aspartate transferase (AST)	84 U/L	13-41 U/L
Alkaline phosphatase (ALK)	259 U/L	42-119 U/L
Lipase	90 U/L	9-50 U/L
Brain natriuretic peptide (BNP)	109 pg/ml	<100 pg/ml
Troponin	0.004 ng/ml	0.02-0.045 ng/ml

**Figure 1:**
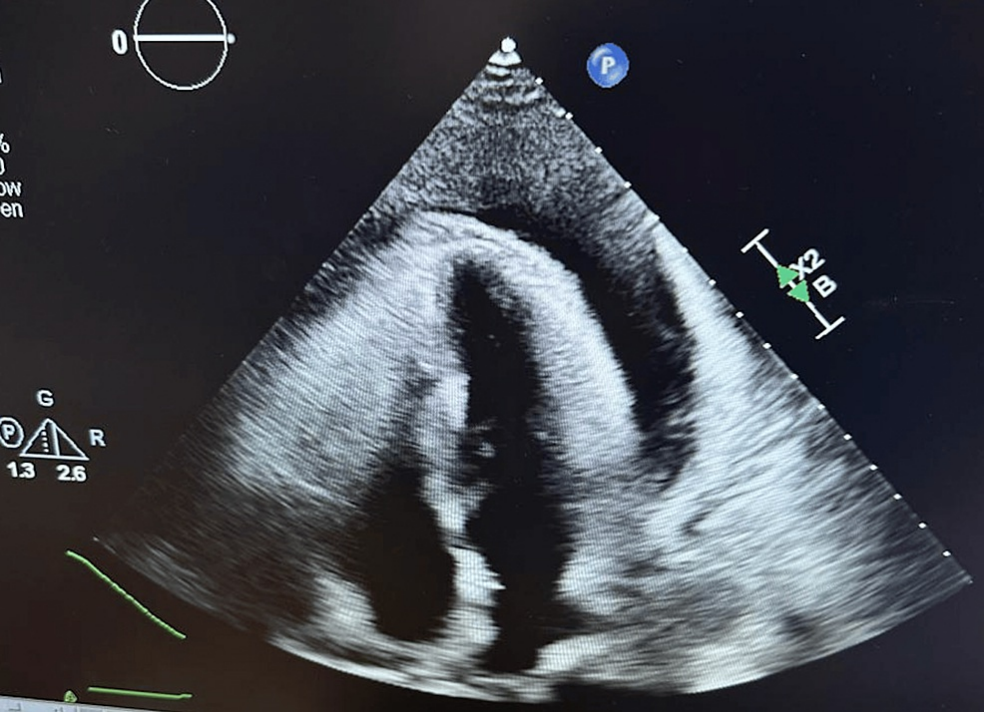
Showing large pericardial effusion compressing the right ventricle

The chest X-ray showed bilateral pleural effusions. The EKG showed sinus rhythm and low-voltage QRS complexes. Stat 2D echocardiogram (Echo) was performed with a normal ejection fraction greater than 70%, grade 1 diastolic dysfunction, and evidence of a large pericardial effusion greater than 2 cm with thickened pericardium and physiology consistent with tamponade, as shown in Figure 1.

Interventional radiology (IR) was consulted for emergent pericardiocentesis. Under CT guidance, a catheter was inserted for drainage, and 250 milliliters (ml) of hemorrhagic fluid were removed and sent for diagnostic studies. The patient was transferred to the intensive care unit (ICU) for close monitoring. The etiology of cardiac tamponade was attributed to malignancy due to the patient's prior history of melanoma and a family history of colon cancer. Hence, a diagnostic workup for malignancy was initiated. The patient had a screening colonoscopy, mammogram, and pap smear recently.

The next day, her troponin was elevated at 6.8 nanograms per milliliter (ng/ml), as shown in Table 2.

**Table 2:** Showing elevated troponin

Troponin	Level	
Initial troponin	6.8 ng/ml	0.02-0.045 ng/ml
Repeat troponin	13 ng/ml	0.02-0.045 ng/ml

**Figure 2:**
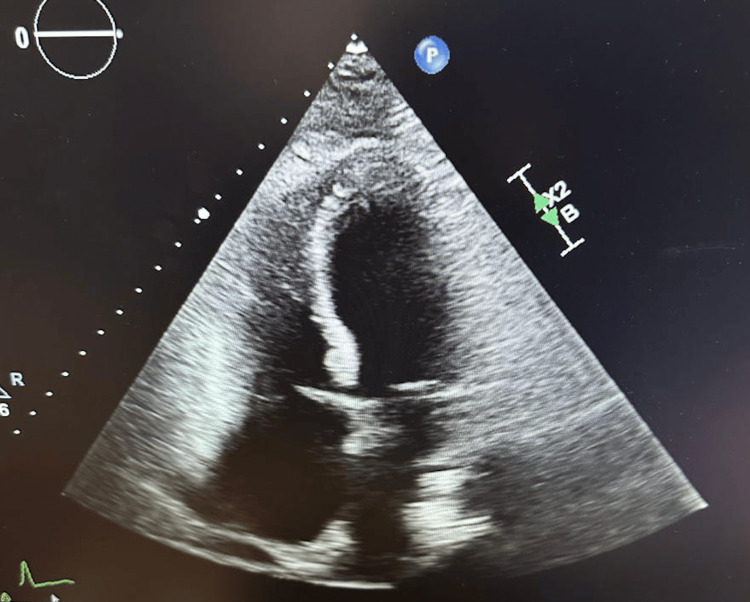
Demonstrating resolution of pericardial effusion after pericardiocentesis

EKG showed no ST or T wave changes. A 2D echo was performed and revealed a decrease in cardiac function with an EF of 40-45%, anterior and inferior wall hypokinesis with sparing of the apex, and a small pericardial effusion <1 cm, as shown in Figure 2.

She was started on Colchicine and Metoprolol. Due to her elevated liver enzymes, a viral hepatitis panel was sent, and an ultrasound was performed, which was within normal limits. The elevated LFTS was thought to be likely from congestive hepatomegaly secondary to pericardial effusion. An autoimmune workup was sent, which was unremarkable except for a positive antinuclear antibody (ANA). Her troponin peaked at 13 ng/mL, as shown in Figure 2, then trended down. A CT abdomen and pelvis was performed as part of the malignancy workup, which revealed gastric wall thickening, cholelithiasis without cholecystitis, and colonic diverticulosis. The patient’s gastric wall thickening was suspicious of possible lymphoma; however, further work was deferred until cytology results. Pericardial drain output continued to decrease over the next few days, and the pericardial drain was removed.

Repeat echo after pericardial drain removal showed an ejection fraction (EF) of 35-40% and no pericardial effusion. Empagliflozin and furosemide were started due to low cardiac function, and spironolactone was added over the next few days. The low EF was attributed to stress-induced Takotsubo cardiomyopathy. Pericardial fluid studies were consistent with exudative etiology, and cytology results revealed large atypical lymphoid cells with CD20, CD79A, and BCL6 markers consistent with the diagnosis of diffuse large B cell lymphoma. The patient's symptoms continued to improve with treatment. She was discharged to home with recommendations for an outpatient positron emission tomography (PET) scan for staging and follow-up with oncology for diffuse large B cell lymphoma treatment.

On outpatient follow-up, the patient remained compliant with her medications, with a resolution of abdominal distention and bilateral leg swelling. A repeat echo one month later revealed an improved EF of 60%. 

## Discussion

Diffuse large B-cell lymphoma (DLBCL) is the predominant subtype of non-Hodgkin lymphomas (NHL), constituting approximately 25% to 30% of all cases [3]. In instances of primary cardiac lymphoma (PCL), DLBCL emerges as the most frequently encountered NHL on histological examination [3]. While the incidence of cardiac and pericardial involvement by lymphoma is notably rare, accounting for just 0.5% of cardiac involvements and 1% of extranodal NHL presentations, it typically occurs in male patients in their 60s [3]. In contrast, the patient in our case is an elderly woman, highlighting the atypical presentation of DLBCL with pericardial effusion and tamponade without primary bone marrow involvement [4]. This emphasizes the necessity of recognizing that DLBCL can manifest spontaneously with pericardial effusion.

Primary cardiac lymphomas are specifically defined as lymphomas confined to the heart and pericardium [4]. These malignancies typically affect the right heart, particularly the right atrium, although literature reports a few instances where the bulk of the lymphoma is located in the left heart sections [3]. In our reported case, the patient presented with a significant pericardial effusion (>2 cm) leading to cardiac tamponade, with no direct tumor involvement of the heart tissue. Diagnostic and staging procedures heavily rely on multimodality imaging, including transthoracic echocardiograms (TTE), CT scans, MRIs, and PET-CT, which are crucial for accurately characterizing these lesions [4]. Among patients with PCL, only 20% display pericardial tamponade at initial presentation, commonly presenting with symptoms such as dyspnea and arrhythmia [4].

This case is particularly illustrative of DLBCL's ability to present with cardiac tamponade, especially within immunocompromised populations who are at greater risk for spontaneous pleural effusion and tamponade due to DLBCL. The patient’s medical history includes melanoma and Crohn’s disease, likely contributing to an immunocompromised state. Notably, survivors of cutaneous melanoma exhibit a significantly elevated risk of developing autoimmune disease, which predisposes them to develop non-Hodgkin lymphoma [5]. DLBCL is also recognized as an AIDS-defining illness, complicating HIV infection. For cancer patients with malignant pericardial effusion, pericardiocentesis with extended drainage is a viable therapeutic option, characterized by low morbidity and complication rates. Furthermore, the administration of low-dose colchicine post-pericardiocentesis has demonstrated beneficial effects on clinical outcomes [6].

Treatment responsiveness varies within DLBCL subtypes; patients with Germinal Center B-cell-like (GCB) DLBCL generally respond well to six cycles of the R-CHOP regimen (rituximab, cyclophosphamide, doxorubicin, vincristine, and prednisone) administered every 21 days [7]. Conversely, patients with activated B-cell-like (ABC) DLBCL, particularly those with double-hit and triple-hit lymphomas, exhibit poorer outcomes with R-CHOP [7]. Such patients are frequently enrolled in clinical trials exploring the efficacy of R-CHOP combined with agents like lenalidomide, bortezomib, or ibrutinib. For more aggressive cases, a dose-adjusted regimen of etoposide, doxorubicin, and cyclophosphamide with vincristine and prednisone in combination with rituximab (EPOCH-R) is indicated [7].

## Conclusions

It is imperative to recognize the potential for DLBCL to present as pericardial effusion leading to cardiac tamponade. In such scenarios, immediate bedside echo should be performed, followed by interventions like pericardiocentesis with extended drainage, supplemented by low-dose colchicine, which has been proven beneficial.
